# Comparison of shock wave lithotripsy (SWL) and retrograde intrarenal surgery (RIRS) for treatment of stone disease in horseshoe kidney patients

**DOI:** 10.1590/S1677-5538.IBJU.2015.0023

**Published:** 2016

**Authors:** Mehmet Ilker Gokce, Zafer Tokatli, Evren Suer, Parviz Hajiyev, Aykut Akinci, Baris Esen

**Affiliations:** 1 Department of Urology, Ankara University School of Medicine, Ankara, Turkey;; 2 Department of Urology Doruk Hospital Ankara, Turkey

**Keywords:** Lithotripsy, Surgical Procedures, Operative, Fused Kidney, Therapeutics

## Abstract

**Objectives:**

In this study it is aimed to compare the success and complication rates of SWL and RIRS in treatment of HSK stone disease.

**Materials and methods:**

In this retrospective study data of 67 patients treated with either SWL (n=44) or RIRS (n=23) for stone disease in HSK between May 2003 to August 2014 was investigated. age, gender, stone size and multiplicity, stone free status, renal colic episodes and complication rates of the SWL and RIRS groups were compared.

**Results:**

Mean age of the population was 42.5±8.2 (range: 16-78) years and mean stone size was 16.9±4.1 mm. SWL and RIRS groups were similar with regard to demographic characteristics and stone related characteristics. SFR of the SWL and RIRS groups were 47.7%(21/44 patients) and 73.9% (17/23 patients) respectively (p=0.039).Renal colic episodes were observed in 3 and 16 patients in the RIRS and SWL groups respectively (p=0.024). No statistically significant complications were observed between the SWL (8/44 patients) and RIRS (4/23) groups (p=0.936).

**Conclusions:**

In HSK patients with stone disease, both SWL and RIRS are effective and safe treatment modalities. However RIRS seems to maintain higher SFRs with comparable complication rates.

## INTRODUCTION

Renal anomalies are associated with increased rates of stone disease and the horseshoe kidney (HSK) is the most common renal fusion anomaly. It is observed in approximately 1 in 400 to 1 in 666 births (1-3). This anomaly leads to anterior displacement of the renal pelvis and associated high insertion of the ureter. This anatomical abnormality causes impaired drainage of the collecting system and urinary stasis and concomitant stone formation (2).

Percutaneous nephrolithotomy (PCNL), shock wave lithotripsy (SWL) and retrograde intrarenal surgery (RIRS) are the treatment modalities of choice for HSK stones. PCNL maintains high success rates but it is associated with higher complication rates therefore the latter two alternatives are commonly applied. RIRS is being increasingly used in the treatment of stone disease particularly in HSK patients with holmium laser lithotriptors (4, 5).

Success rates in HSK patients after SWL is highly variable and stone free rates (SFR) of 31-100% were reported in the literature (6-11). SFR of RIRS in the management of HSK patients were reported to be 70% and 88.2% in the two recently published studies (4, 5). However the current literature lacks studies comparing the success rates and complication rates of SWL and RIRS in treatment of HSK stone disease and in this study it is aimed to compare these two treatment modalities in terms of SFRs and complication rates.

## PATIENTS AND METHODS

Data of 67 patients treated with either SWL or RIRS for stone disease in HSK between May 2003 and August 2014 was investigated retrospectively. Stone disease was diagnosed by use of renal ultrasonography (USG), plain abdominal radiography and intravenous urography (IVU) or non-contrast enhanced computarized tomography (NCT). Appropriate antibiotic therapy was prescribed prior to SWL or surgery in case of diagnosed urinary tract infection. Demographic and stone related characteristics collected were: age, gender, stone size, localization and multiplicity and duration of hospitalization for RIRS group.

SWL was performed with ELMED Complit SWL device (Elektronik ve Medikal Sanayi ve Ticaret A.S, Ankara,Turkey). All patients were treated on an outpatient basis without anesthesia but sedation was applied with midozolam 0.1mg/kg intravenously when the patient could not tolerate the procedure. All treatment sessions were limited to 3000 shocks with frequency of 60-120shocks/minute and shock wave intensity was started at 14 kV and gradually increased to 21 kV. None of the patients were stented prior to the procedure.

RIRS procedure was performed with the patient under general anesthesia. The patient was positioned in lithotomy position and in a slight Trendelenburg position to allow the stone fragments to fall into the more upper calices. A 22F cystoscopy was introduced to visualize the ureteral orifice. Ureteral baloon dilation was performed when necessary and a hydrophilic guidewire was introduced. Next a ureteral access sheath, of various sizes (9.5/11.5 F or 12/14F Flexor (Cook Surgical, Indianapolis, IN)) was placed and then the surgeon passed the endoscope into the renal collecting system. Automatic flow irrigation at a pressure of 100cm H_2_O associated with the manual pump was used to improve visualization. The laser energy was 0.8-1.2 J and frequency 8-12 Hz. All the lower pole stones were repositioned to the upper calices. The procedure was ended when there was no visible fragments ≥3mms in direct vision or under fluoroscopy. Double J stent was placed routinely after the procedure. A Foley catheter was inserted and taken out after 12-24 hours to ensure maximal drainage.

Complication rates during the perioperative period for both SWL and RIRS were recorded. All patients were evaluated by plain radiography and either renal USG or NCT at 1-6 weeks after SWL and 2-6 weeks after RIRS to evaluate the SFRs. Stone free status was defined as no residual fragments ≥3mm in size. Renal colic episodes in the postoperative period were recorded and the two treatment modalities were compared for success and complication rates. Macroscopic hematuria was accepted as a complication rather than microscopic hematuria.

Statistical analysis was performed with SPSS ver. 20.0. Chi square test was used to compare categorical variables and Student t-test was applied for continuous variables of the treatment groups. For statistical significance p value of 0.05 was accepted.

## RESULTS

A total of 52 stones in 44 patients and 32 stones in 23 patients were treated with SWL and RIRS respectively. Mean age of the population was 42.5±8.2 (range: 16-78) years and mean stone size was 16.9±4.1mm (range: 6-25mm). SWL and RIRS groups were similar with regard to demographic characteristics and stone related characteristics and the results are summarized in Table-1. Mean duration of hospitalization was 1.8 days (1-3 days) in the RIRS group. Median number of SWL sessions was 3 (range: 1-6).

**Table 1:** Demographic characteristics of the groups.

Parameters	SWL group (n=44)	RIRS group (n=23)	P value
Age (mean±SD)	42.8±8.4	44.2±9.9	0.17
**Gender**			0.62
Male (%)	32 (72.7)	18 (78.3)	
Female (%)	12 (27.3)	5 (21.7)	
Stone size, mm (mean±SD)	16.8±4.4	17.1±5.1	0.27
Multiple stones (%)	8 (18.1)	9 (39.1)	0.06
**Stone location**			0.917
Lower pole	12 (27.2)	6 (26.1)	
Pelvis and upper pole	32 (72.8)	17 (73.9)	

SFR of the SWL and RIRS groups were 47.7% (21/44 patients) and 73.9% (17/23 patients) respectively (p=0.039). In the SWL group, 10 patients (22.7%) achieved stone free status after a single session of SWL. Renal colic episodes were observed in 3 and 16 patients in the RIRS and SWL groups respectively (p=0.024). The results are summarized in Table-2. Double J stent placement was required in 13 patients (29.5%). SFR of the patients in SWL group with or without double J stent placement was found to be similar (6/13 patients vs 15/31 patients, p=0.892). Similarly double J stent placed had no effect on prevalence of renal colic episodes (5/13 patients vs 11/31 patients, p=0.851).

**Table 2:** Treatment results of the groups.

Parameters	SWL group (n=44)	RIRS group (n=23)	P value
SFR (%)*	21 (47.7)	17 (73.9)	0.039
Renal colic episode (%)	16 (36.3)	3 (13)	0.024
Complications	8 (18.1)	4 (17.4)	0.936

*SFR: Stone free rate

When the complication rates of the two groups were compared, no statistically significant complications were observed between the SWL (8/44 patients) and RIRS (4/23) groups (p=0.936). Hematuria was the most common complication and it was observed in 6 and 3 patients in the SWL and RIRS groups respectively. Fever was observed in 1 patient in each group and perirenal hematoma was observed in 1 patient in the SWL group. Results of the complication rates are summarized in Table-2.

## DISCUSSION

HSK is the most common renal fusion anomaly and the abnormal position of the kidney and unusual course of the upper ureter over the isthmus not only stands as a cause for stone formation but also makes the stone disease treatment more challenging. PCNL has already proved its efficacy on large stones located in HSK but the complicated nature of this method and vulnerability to complications (complication rates 14.3-29.2%) makes SWL and RIRS more feasible options (12-14).

Efficacy of SWL in stone disease of HSK has been studied since 1989 (15) and variable success rates have been reported. SFRs up to 100% were reported in the small sample sized studies (10). Differences in success rates depend on the definition of success, number of SWL sessions and duration of follow-up intervals. Sheir et al. reported 71.4% SFR in their series of 49 patients (9). However, they did not mention data on number of treatment sessions. In another series of 50 patients, 29 patients were available for follow-up and 75.9% SFR was reported (16). An important point in this study is the exclusion of patients with hydronephrosis, delayed drainage in radionuclide scans which takes into mind the selection bias resulting in high success rate with relatively low number of sessions (mean 1.1 sessions). In our cohort, SFR was achieved in 47.7% of the patients after a median of 3 SWL sessions.

Ray et al. reported their series of 61 stones in 41 patients to identify the determinants of SWL success in HSK patients. They argued on incremental benefit of more than 2 sessions of SWL due to the discordance of high stone fragmentation rate (63.6%) and relatively low stone clearance rate (39.1%). However in this study the primary outcome measure was the single session success rate and any patient with more than one SWL sessions was accepted as treatment failure (17). In our study, success rate was not determined based on single SWL session but only 12 patients (21.4%) achieved stone free status after a single session of SWL.

There are a relatively low number of studies in the current literature on success rates of RIRS in HSK patients compared to studies on SWL and PCNL. In the normally located kidneys, RIRS is increasingly used all over the World with potentially higher success rates compared to SWL and lower complication rates compared to PCNL. Therefore not only reporting success and complication rates of RIRS in HSK patients but also comparison of treatment modalities is of importance in this specific patient population. However to our knowledge there is no study published comparing RIRS and SWL.

First series of RIRS in HSK patients was published in 2005 and stone clearance was achieved in 3 of the 4 patients (18). Following that, two larger series were published. In the first study, Molimard et al. reported their experience in 17 patients and SFR was achieved in 15 patients (88.2%) with mean stone size of 16mm. The success rate was comparable to PCNL and better than SWL studies with no major complications. However, the results were achieved by a highly experienced surgeon (4). In the same study 7 (41.2%) patients required more than one session of RIRS. In the second study, 25 renal stones of 20 patients were treated with RIRS and SFR of 70% was reported. The authors mentioned their success rates comparable with PCNL and better than SWL with the advantage of low complication rates (5).

Our study involves the highest number of HSK patients (32 stones in 23 patients) that underwent RIRS. In our study SFR of 73.9% was achieved with acceptable complication rates (4 of the 23 patients) which is comparable to previously published series (4, 5). When the results of SWL and RIRS are compared, significantly higher success rates were achieved with RIRS. Additionally renal colic episodes were observed more frequently in SWL group. One of the main advantages of RIRS compared to SWL is the repositioning of the lower pole stones to upper calices and this may facilitate the stone clearance rates following fragmentation. The main disadvantage of RIRS is the need for general anesthesia. However no major complication related to anesthesia was observed in the current study. Also, no significant difference was observed with regard to complication rates, hematuria being the most common complication in both groups.

The major limitation of the study is the retrospective nature and lack of randomization. Also, the procedures were performed by 4 different surgeons with variable level of experience. Also, neither duration of follow-up nor method of imaging in the preoperative and postoperative period was standardized.

## CONCLUSIONS

In HSK patients with stone disease, both SWL and RIRS are effective and safe treatment modalities. However, RIRS seems to maintain higher SFRs with comparable complication rates. To determine the best method for treatment of stone disease in HSK patients, randomized trials comparing SWL, RIRS and PCNL are needed.
